# Assessment of coconut water added to *Numida meleagris* egg yolk as cryoprotectant for goat semen

**DOI:** 10.1590/1984-3143-AR2021-0114

**Published:** 2022-09-26

**Authors:** Laércio Fontinele Bandeira de Macêdo, Letícia Soares de Araújo Texeira, Clarissa de Castro e Braga, Kenney de Paiva Porfírio, Sara Camila da Silveira Costa, Louis Henrique Miyauchi Silva, Wcleuden Matias Nascimento, Francisca Kelly dos Santos Silva, Nildene Silva Andrade Bandeira, Rômulo José Vieira, Ana Lys Bezerra Barradas Mineiro, Cristiane Clemente de Mello Salgueiro, José Ferreira Nunes, Janaina de Fátima Saraiva Cardoso, Ney Rômulo de Oliveira Paula

**Affiliations:** 1 Departamento de Clínica e Cirurgia Veterinária, Universidade Federal do Piauí, Teresina, PI, Brasil; 2 Centro Universitário Internacional, Teresina, PI, Brasil; 3 Faculdade de Veterinária, Universidade Estadual do Ceará, Fortaleza, CE, Brasil

**Keywords:** goat, semen, cryoprotectant, egg yolk

## Abstract

Effects were assessed of the dilutants TRIS and ACP - 101c® with the addition of different guinea fowl (*Numida meleagris*) egg yolk concentrations. Fifteen ejaculates were collected from five goats of the Anglo Nubian breed. The ejaculates were pooled and then divided into 12 groups, two control groups (GC1 TRIS, with 2.5% *Gallus gallus domesticus* hen egg yolk GOGD), (GC2 Control Group ACP - 101c®, with the addition of 2.5% *Gallus gallus domesticus* hen egg yolk GOGD) and ten experimental groups (EG), containing TRIS and ACP added with different concentrations of egg yolk from guinea hen (*Numida meleagris*) (TRIS 2,5% GONM; TRIS 5% GONM; TRIS 10% GONM; TRIS 15% GONM; TRIS 20% GONM; ACP® 2,5% GONM; ACP® 5% GONM; ACP® 10% GONM; ACP® 15% GONM; ACP® 20% GONM). Then cryopreservation was carried out and the samples stored in liquid nitrogen (-196 °C). After seven days, the samples were thawed and assessed for spermatic kinetics, immunofluorescence and sperm morphology. Analysis of GOMN by the CASA system showed that the various parameters were similar to those of GOGD (P>0.05). The membrane integrity, mitochondrial potential and the acrosome were not influenced by the treatment (P>0.05) nor by the dilutant used for cryopreservation (P>0.05). The spermatic morphology was also preserved by the different GOGD and GONM concentrations in the ACP® and TRIS dilutants, with no statistically significant differences (P<0.05). It was concluded that *Numida meleagris* egg yolk, as external membrane cryoproctant added to the dilutants ACP-101c® and TRIS, improved goat semen quality.

## Introduction

It is known that several dilutant media have already been elaborated to confer energy and protection to spermatozoids against cryoprotective injuries and to maintain a suitable environment for their survival. These used media present in their composition a variety of different substances (Purdy, 2006).

Furthermore, studies continue to be developed to discover new dilutant media that better protect the sperm cells during the stages of cryopreservation. And, in this context, coconut water has been listed as a satisfactory component for this objective, because it is a sterile, not very acid, solution consisting of neutral fats inducers or growth factors that stimulate cell division and various electrolytes, conferring coconut water a density and pH compatible with blood plasma. It has also been reported that coconut water supplies nutrients necessary for the survival and viability of male and female gametes during the cryopreservation process (Carvalho et al., 2006).

Research has shown satisfactory results for using *in natura* coconut water-based dilutants for sperm cell preservation of several animal species including dogs (Cardoso et al., 2003), sheep (Machado et al., 2006) and goats (Salgueiro et al., 2002). Based on these studies, powdered coconut water (ACP®) was developed, and has been viable as dilutant medium for semen cryopreservation in the goat species (Salgueiro et al., 2003).

In this perspective, considering the development of more studies in the area of cryopreservation of goat semen, in order to obtain satisfactory results, it is worth inferring that the egg yolk of *Numida meleagris* constitutes a valuable component to be present in seminal extenders, because it has a rich composition: crude protein 15.74%, amino acids (glutamine 13.9%, aspartate 9.63%, leucine 8.07%, arginine 7.12%, lysine 7.01%, valine 5.61% , proline 5.6%, glycine 5.6%, phenylalanine 5.56%, isoleucine 5.03%, serine 4.94%, alanine 4.7%, threonine 3.95%, tyrosine 3.69%) (Adeyeye, 2010). In addition, the egg yolk of *Numida meleagris* (Angola chicken) has a higher concentration of minerals: potassium, iron and calcium when compared to that found in the egg yolk of *Gallus gallus domesticus* and in the egg yolk of *Gallus gallus hybrid*. This fact is relevant, since minerals are essential for sperm viability (Adeyeye, 2010).

It is pointed out, regarding the dilutant media, that hen egg yolk (*Gallus gallus domesticus*) is the ingredient most used for domestic mammal species, giving protection to male gametes submitted to the freeze-thaw process (Forouzanfar et al., 2010). But the guinea fowl (*Numida meleagris*) egg yolk has not been assessed in studies related to dilutant media for domestic mammal semen.

Thus the objective of the present study was to assess the viability *in vitro* of goat semen cryopreserved in powdered coconut water-based medium (ACP-101c®) with the addition of an alternative cryoprotectant, guinea fowl (*Numida meleagris*) egg yolk.

## Methods

### Ethics in animal experimentation

The project was assessed and approved by the Commission for Ethics in the Use of Animals at the State University of Piauí, protocol number 07614-2018 on 20.07.2018. For scientific research purposes, it is in compliance with Law N° 11.794. of October 8, 2008. Decree N° 6.899 of July 15 2009, and with the norms published by the National Council of Control in Animal Experimentation.

### Animals used in the research

The research was carried out in the animal reproduction sector at the Federal University of Piauí, *Campus* da Socopo, Teresina, Piauí, Brazil, geographic coordinates: 5º 03' 23.1’’ latitude south and 42º 47’ 27.9’’ longitude west, 72.2 meters mean altitude.

5 reproducers were used of the goat species, Anglo Nubian breed, pure bred, average age 4 years old, clinically healthy and with 3.5 body condition score on a scale of (0-5). The animals used came from a farm located in the municipality of Campo Maior, in the state of Piauí, geographic coordinates: latitude 4°51’35.75’’ S and longitude 42°12’47.18’’W, 129 meters mean altitude. The animals were fed daily with bulk (*Pennisetum purpureum Schum*), commercial concentrate (20% protein pelletized feed, 300 g/animal/day), mineral salt specific for goats (Caprinofós®) and water ad libertum.

The reproducers underwent the reproductive clinical exam to assess their general health and that of the reproductive tract, following criteria established by the Brazilian College of Animal Reproduction (CBRA, 2013).

### Chemical evaluation of Numida meleagris egg yolk

Guinea fowl *Numida meleagris* eggs were used from free range fowl with laying at average five-day intervals, from rual producers in the Campo Maior, Piauí microregion. The birds were fed seeds, insects, corn kernels and native grass dry matter (*Axonopus purpusii Chas*) and other species *Mésosetum, Axonopus, Pás palum, Panicum* and *Eriochloqde*. They also received energy and protein concentrates in solid form in a trough and water was available ad libertum.

The bromatological analyses were carried out at the Federal University of Piauí, in the Health Science Center, Nutrition Department, in the Food Bromatology and Biochemical Laboratory and in the Laboratory of Product Development and Food Sensorial Analysis Laboratory.

The dry matter, ash, ether extract, lipids, carbohydrates and proteins were analyzed following methodology described by the Official Association of Analytical Chemistry (AOAC, 2006).

Moisture was determined by gravimetry, by the method of drying in a chamber at 105 ºC. Ash was determined by incineration in a mufla oven at 550 ºC. The lipids (corresponding to the stereo extract fraction) were obtained by the intermittent oil and fat extraction method, in a Soxhlet extractor, using petroleum ether solvent. Proteins were determined by the macro Kjeldahl method, where the 6.25 factor was used to convert the total nitrogen content to protein. The carbohydrate content was obtained by the difference of the other of the centesimal composition constituents (moisture, ash, lipids and proteins).

The calorie value of the “nugget” under study was calculated using ATWATER conversion factors: 4 kcal/g for proteins, 4 kcal/g for carbohydrates and 9 kcal/g for lipids (Watt and Merrill, 1963).

Later, the total carotenoid contents were determined following methodology by Alvarez-Suarez et al. (2011), using acetone/hexane solvent and all the extracts were elaborated following the methodology proposed by Rufino et al. (2010).

Lastly, the antioxidant activity was determined by the radical capture method, using the DPPH radical (2.2- diphenyl-1-picrylhydrazyl).

### 2.4. Semen collection and sperm evaluation

Fifteen semen collections were taken from each reproducer. First the reproducer foreskin was thoroughly cleaned with 0.9% physiological solution and paper towel. The collections were made by the artificial vagina method, specific for small ruminants, heated to 38ºC attached to a Flacon-type graded collecting tube. One doe with induced estrus was used during the collections. After each collection, the ejaculate was protected from direct sunlight, take to the laboratory and kept in a water bath at 37 ºC (CBRA, 2013; Qin et al., 2018).

Next the subjective macroscopic assessments of the ejaculate were made, including volume, color and aspect. The sperm parameters were assessed sequentially: turbulence, motility, vigor, sperm morphology and sperm concentration (CBRA, 2013).

An optical, biological and binocular microscope was used at 40x magnification (Microscope Binocular 40-1000X 2.0MP USB Camera Digital Microscope 100 pcs) to assess total motility (scale of 0-100%) and sperm vigor (0-5), a drop of semen was placed between the previously warmed slide and slide cover and kept at 37 ºC. For the sperm morphology analysis, 200 sperms were counted per stained slide, under an optical microscope, 400x magnification.

To determine the sperm concentration, the semen was diluted in formalin-saline (1: 200) solution, and counted in a Neubauer chamber under a 400x magnification microscope (CBRA 2013; Qin et al., 2018).

### Formation of experimental groups

The ejaculates collected were pooled and the sperm concentration was assessed in a Neubauer chamber. The dilution rate was adjusted to a final concentration of 320 million spermatozoids/mL.

The semen collected was united, forming a pool. Subsequently, the pool was divided in to twelve equal volume aliquots, with at least five milliliters semen and diluted in the dilutant media for cryopreservation, TRIS and ACP®. Next, the following control groups were formed: two control groups (CG), with the addition of domestic hen (*Gallus gallus domesticus*) egg yolk GC1 –TRIS 2.5%GOGD and GC2 – ACP® 2.5%GOGD and ten experimental groups EG, with the addition of guinea hen egg yolk Numida meleagris - TRIS 2.5% GONM; TRIS 5% GONM; TRIS 10% GONM; TRIS 15% GONM; TRIS 20% GONM; ACP® 2.5% GONM; ACP® 5% GONM; ACP® 10% GONM; ACP® 15% GONM; ACP® 20% GONM.

### Semen Freezing-Thaw Process

Eight 0.25 mL French well plates (IMV Technologies, Campinas, São Paulo, Brasil) were filled for each group.

The cryopreservation process was carried out with the TK3000® apparatus (TK Tecnologia em Congelação Ltda., Uberaba, Brazil), following the manufacturer’s instructions, using a fast curve (0.5 ºC/min. to 5 ºC; 15 ºC/min. to -20 ºC and 10 ºC/min. to -120 ºC). Later the samples were immersed in liquid nitrogen (-196 ºC) and stored on labeled racks in cryopreservation bottles.

### Analysis of sperm kinetics after thawing

The post-thawing sperm kinetics were analyzed at the Laboratory of Goat and Sheep Semen Technology, in the Integrated Biotechnology Nucleus, of the Veterinary Medicine Faculty, at the Ceará State University, Fortaleza, Ceará, Brazil, by the CASA system (Computer-assisted Sperm Analyzer), using the Software Sperm Class Analyser® (SCA) (Microptics, SL, version 3.2.0. Barcelona, Spain).

After a minimum period of 7 days, the samples were thawed in a water bath at 37 °C for 30 seconds, placed in Eppendorf type microtubes and homogenized for immediate analysis (Arangasamy et al., 2018).

To analyze the cryopreserved samples in medium containing TRIS/qy, a 10 μL thawed semen sample was diluted in 50 μL TRIS medium [3.786 g Tris (hydroxymethyl) aminomethane; 2.11 g citric acid; 1.0 g fructose; 100 mL distilled water] previously heated in a water bath to 37 ºC. While for the samples cryopreserved in ACP-101c®-based medium, one 10 μL thawed semen sample was diluted in 50 μL ACP-101c medium (3.25 g; 50 mL distilled water).

Next, 10 μL of these dilutions were placed in a Makler chamber (Sefi Medical Instruments Ltda., Haifa, Israel), previously heated to 37 ºC and assessed under a contrast phase microscope coupled to a video camera adapted to the system (Nikon™ H5505. Eclipse 50i, Japan).

The following program was used to evaluate the movement characteristics of frozen-thawed sperm: 30 μm/s; limit for medium speed: 60 μm/s; minimum straightness for progressive spermatozoids: 80%, captured in three fields. A total of 200 spermatozoids were assessed by the CASA system in each sample.

The parameters included: total motility (TM - %), progressive motility (MP), curvilinear speed (VCL - μm/s), mean trajectory speed (VAP - μm/s), linear speed (VSL - μm/s), straightness (LIN - %), amplitude of the lateral head displacement (ALH - μm), oscillation or straightness index (STR - %) and individual cross beat frequency (BCF - Hz) for each spermatozoid cell.

### Immunofluorescence analysis after thawing

#### Acrosome integrity analysis

The acrosome integrity was analyzed using the conjugated fluorescein isocyanate probe (FITC-PNA; Sigma-Aldrich®, St. Louis, MO, USA). The spermatozoids were classified as integral acrosome carriers (AI) when the acrosome region presented intense green fluorescence. They were considered non-integrated acrosome when intense green fluorescence was not present or when the fluorescent green was restricted to the sperm head equatorial region, according to the technique described by (Roth et al., 1998).

#### Analysis of plasma membrane integrity

The analysis of the plasmatic membrane integrity (IMP) was analyzed using an association of two fluorescent probes: Carboxyfluorescein diacetate (DCF; Sigma-Aldrich®, St. Louis, MO, USA) (0.46 mg/ml in PBS) and Propidium Iodide (IP; Sigma-Aldrich®, St. Louis, MO, USA) (0.5 mg/ml in PBS). After preparing the slide with the fluorescent markers, the spermatozoids were assessed under a fluorescence microscope in a controlled illumination room (magnification 100x). One hundred spermatozoids were assessed under fluorescence microscope, that were classified as integral, when the spermatozoid head was colored green, and damaged, when red color was observed (Harrison and Vickers, 1990).

#### Mitochondrial activity analysis

The mitochondrial activity was determined by the technique proposed by (Guthrie and Welch, 2006) using a lipophilic cationic Fluorochrome probe (JC-1; Sigma-Aldrich®, St. Louis, MO, USA). The orange stained cells were classified with high mitochondrial potential, while those stained green were classified with low membrane potential.

### Statistical analysis

Means and standard deviations were obtained and analysis of variance (ANOVA) was carried out followed by the Fisher PLSD test for the parameters sperm motility, morphology, sperm vaibility analyses, using fluorescent probes and sperm kinetics (CASA) and the Kruskal-Wallis test was used to determine the parameter sperm vigor.

The means were compared by the Tukey test, according to the coefficient of variation obtained, considering a 5% level of significance. The PROC GLM was used (General Linear Models) of the Software SAS® (Statistical Analysis System) for Windows version 9.0.

## Results

### Chemical evaluation of the egg yolk of Numida meleagris

Before using the egg yolk of Numida meleagris, a bromatological evaluation of the chemical composition of the yolk was performed, and it was possible to identify the parameters shown in Table 1.

**Table 1:** Chemical composition of Angolan chicken egg yolk (*Numida meleagris*).

**Parameters**	**Values found**
moisture (%)	51.84
Crude protein (%)	9.37
Antioxidant Activity (DPPH) µmol/100g	347.88
Gross ash (%)	1.94
pH	6,99
Carotenoids (mg/100g)	2.541
Beta-carotene (mg/100g)	52.37
Lipids (%)	34.56
Carbohydrates (mg/100g)	2.29
Valor energético (kcal/100g)	357.68

%: percentage. mg: milligrams. g: grams. DPPH: 2.2-diphenyl-1-picrylhydrazyl. kcal: kilocalorie.

### Subjective evaluation of post-dilution and post-thaw sperm parameters

The macroscopic and microscopic parameters assessed in the fresh semen, immediately after pooling, showed creamy aspect with a 5.63±1.64 mL mean collection volume. This volume was centrifuged to remove the seminal plasma, obtaining 4.97±1.65 mL mean semen volume. The mean motility values after collection were 81.67±10.47, mean vigor 4.0.7±0.70 and mean turbulence 3.93±0.59.

**Figure 1:**
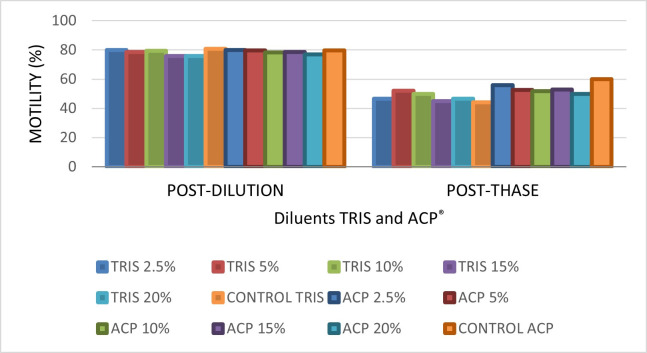
Means ± standard deviation of the sperm parameter (motility) subjective analysis post-dilution and post-thawing of goat semen in different dilutants (TRIS e ACP-101c®) and at different concentrations of egg yolk of the species *Gallus gallus domesticus* (GOGD) and *Numida meleagris* (GONM). Prepared by the authors (2021).

Figure 1 shows the percentage of sperm cells in terms of motility after the processes of dilution, freezing and thawing of goat semen.

Regarding comparison among the different concentrations of *Numida meleagris* egg yolk of the experimental groups and the *Galus galus domesticus* egg yolk of the control group (ACP® 2.5%), no difference was observed regarding the yolk origin (P > 0.05).

Table 2 shows the results of the subjective analysis and the spermatic kinetics post dilution and pos- thawing for the parameter vigor of goat semen diluted in TRIS and ACP-101c® with the addition of *Galus galus domesticus* egg yolk (control group) and *Numida meleagris* egg yolk (experimental groups).

**Table 2:** Means (± d.p.) of the sperm parameter (vigor) post dilution and post-thawing of goat semen in different dilutant (TRIS and ACP-101c®) and in different concentrations of egg yolk from the species *Gallus gallus domesticus* (GOGD) and *Numida meleagris* (GONM).

**Experimental Groups**	**Post dilution** **Vigor (0-5)**	**Post-thawing** **Vigor (0-5)**
G.C2. ACP® GOGD 2.5%	4.03 ± 0.77^a^	3.27 ± 0.80^b^
G.E. ACP® GONM 2.5%	3.83 ± 0.70^a^	2.93 ± 0.80^b^
G.E. ACP® GONM 5%	3.80 ± 0.94^a^	3.13 ± 0.92^a^
G.E. ACP® GONM 10%	3.83 ± 1.03^a^	3.13 ± 0.92^b^
G.E. ACP® GONM 15%	3.63 ± 1.01^a^	3.27 ± 0.88^a^
G.E. ACP® GONM 20%	3.60 ± 0.91^a^	3.20 ± 0.94^a^
G.C1. TRIS GOGD 2.5%	3.87 ± 0.83^a^	2.93 ± 0.70^b^
G.E. TRIS GONM 2.5%	3.60 ± 1.06^a^	2.93 ± 0.88^b^
G.E. TRIS GONM 5%	3.60 ± 0.83^a^	2.87 ± 0.92^b^
G.E. TRIS GONM 10%	3.43 ± 0.86^a^	2.93 ± 1.03^b^
G.E. TRIS GONM 15%	3.63 ± 0.94^a^	2.60 ± 1.12^a^
G.E. TRIS GONM 20%	3.63 ± 0.94^a^	2.80 ± 1.08^a^

Different letters among columns indicate significant statistical differences (P<0.05). Significant statistical differences were not observed (P>0.05) between lines in the same column. G.C2. ACP®2.5% = Control group containing 2.5% of egg yolk of *Gallus gallus domesticus.* G.E. ACP®2.5%= Experimental group containing 5% of egg yolk of *Numida meleagris* (GOGD). G.E. ACP®5%= Experimental group containing 5% of egg yolk of *Numida meleagris* (GONM). G.E. ACP®10%= Experimental group containing 10% of egg yolk of *Numida meleagris* (GONM). G.E. ACP®15%= Experimental group containing 15% of egg yolk of *Numida meleagris* (GONM). G.E. ACP®20%= Experimental group containing 20% of egg yolk of *Numida meleagris* (GONM). G.C1. TRIS 2.5% = Control group containing 2.5% of egg yolk of *Gallus gallus domesticus* (GOGD). G.E. TRIS 2.5%= Experimental group containing 5% of egg yolk of *Numida meleagris* (GONM). G.E. TRIS 5%= Experimental group containing 5% of egg yolk of *Numida meleagris* (GONM). G.E. TRIS 10%= Experimental group containing 10% of egg yolk of *Numida meleagris* (GONM). G.E. TRIS 15%= Experimental group containing 15% of egg yolk of *Numida meleagris* (GONM). G.E. TRIS 20%= Experimental group containing 20% of egg yolk of *Numida meleagris* (GONM).

In the subjective analysis of post-thawing sperm vigor there was significant difference among the dilutants in the treatments with 5%, 15% and 20% *Numida meleagris* egg yolk (ACP® 75% vs.; P < 0.05). In the treatments with TRIS post-thawing, there was statistical difference in the dilutant in treatments with 2.5% and 10% *Numida meleagris* egg yolk (TRIS 55%; P < 0.05). The groups TRIS 15% and 20% with *Numida meleagris* egg yolk presented the lowest vigor percentages compared to the control group (TRIS 2.5% *Galus galus domesticus* egg yolk) and to the other experimental groups (TRIS 2.5; 5.0 and 10.0% *Numida meleagris* egg yolk) (P < 0.05).

When the ACP-101c® dilutant was used, there was no statistical difference between the yolk origins (*Gallus gallus domesticus* – control group vs. *Numida meleagris*) (P > 0.05). However, assessment of the TRIS dilutant showed a fall in vigor in the groups with 15% and 20% *Numida meleagris* egg yolk (P < 0.05), but the other experimental groups did not show statistical difference in relation to the control (P > 0.05), and it may be considered that the yolk origin was not a predominant factor in the issue.

### Objective evaluation of post-thaw sperm kinetics

In the present study, besides the subjective analysis, a computer aided semen analysis system (CASA) was used because it enabled an objective and precise assessment of the sperm parameters (TM, LIN, VAP, VSL, VCL, STR and OSC).

Table 3 shows the effect of sperm cryoprotectant on goat semen cryopreserved in medium (ACP-101c®) and different concentrations of *Numida meleagris* egg yolk (GONM) and *Galus galus domesticus* egg yolk (GOGD).

**Table 3:** Means (± d.p.) of the sperm kinetic parameters (TM, VCL, VSL and VAP) of goat semen cryoprotectant in ACP-101c® with the addition of different concentrations of *Numida meleagris* egg yolk assessed by CASA post-thawing.

**Experimental Group**	**TM (%)**	**VCL(μm/s)**	**VSL (μm/s)**	**VAP (μm/s)**
GC2-ACP® 2.5%GOGD	21.39 ± 15.36^a^	75.74 ± 14.14^a^	57.90 ± 14.77^b^	64.97 ± 15.32^ab^
ACP® 2.5% GONM	16.25 ± 15.59^ba^	71.71 ± 12.01ª	49.70 ± 12.35^b^	58.33 ± 11.91^a^
ACP® 5% GONM	1.95 ± 8.02^b^	75.69 ±15.20ª	56.68 ± 16.75^b^	65.63 ± 17.34^ab^
ACP® 10% GONM	17.91 ± 13.25^ab^	67.92 ± 13.69^a^	90.35 ± 14.25^a^	61.75 ± 9.69^ab^
ACP® 15% GONM	19.10 ± 16.48^ab^	78.84 ± 9.46ª	60.54 ± 8.67^b^	69.09 ± 9.37^b^
ACP® 20% GONM	16.62 ± 16.61^ba^	79.44 ± 14.97ª	60.19 ± 15.20^b^	68.63 ± 14.80^b^

Different letters among columns indicate significant statistical differences (P<0.05). TM = total motility; VCL = curvilinear speed; VSL = linear speed; VAP = mean trajectory speed. GC2-ACP® 2.5%GOGD = ACP-101c® + 2.5% egg yolk *Gallus gallus Domesticus* (Group control); ACP® 2.5% GONM = ACP-101c® + 2.5% egg yolk *N. meleagris*; ACP® 5% GONM = ACP-101c® + 5% egg yolk *N. meleagris*; ACP® 10% GONM = ACP-101c® + 10% egg yolk *N. meleagris*; ACP® 15% GONM = ACP-101c® + 15% egg yolk *N. meleagris*; ACP® 20% GONM = ACP-101c® + 20% egg yolk *N. meleagris*.

The results obtained for semen cryopreserved in 2.5%, 10%, 15% and 20% GONM were similar to the control group in the total motility parameter (TM). However, the 5% GONM experimental group presented inferior results to those of the control group. In the VCL parameter, significant difference was observed among the experimental groups and the control group. In the VSL the 10% GONM concentrations gave superior results compared to the other groups. For VAP, the control group did not differ from the other treatments. However, the groups containing the concentrations of 15% and 20% GONM showed superior results to those found at the 2.5% concentration.

Table 4 shows the effect of sperm cryopreservation of goat semen in TRIS medium and different GONM and GOGD concentrations, by the computerized analysis system.

**Table 4:** Means (± d.p.) of the sperm kinetic parameters (TM, VCL, VSL e VAP) of goat semen cryoprotectant in TRIS with the addition of different GONM concentrations assessed by CASA post-thawing.

**Experimental Group**	**TM (%)**	**VCL(μm/s)**	**VSL (μm/s)**	**VAP (μm/s)**
GC1-TRIS 2.5%GOGD	13.31 ± 11.96ª	56.06 ± 13.38^ab^	35.35 ± 11.19ª	42.13 ± 12.70ª
TRIS 2.5% GONM	8.59 ± 6.10ª	58.91 ± 12.21^ab^	35.05 ± 11.22ª	41.55 ± 11.71ª
TRIS 5% GONM	11.43 ± 11.0ª	58.93 ± 14.00^ab^	35.27 ± 11.89ª	42.15 ± 12.94ª
TRIS 10% GONM	11.89 ± 8.03ª	64.69 ± 13.91^ab^	35.29 ± 12.31ª	47.45 ± 12.29ª
TRIS 15% GONM	12.25 ± 8.94ª	67.37 ± 8.51ª	38.92 ± 10.63ª	48.03 ± 9.95ª
TRIS 20% GONM	17.61 ± 19.26ª	67.80 ± 12.70ª	37.41 ± 14.11ª	48.40 ± 12.19ª

Different letters among columns indicate significant statistical differences (P<0.05). TM = total motility; VCL = curvilinear speed; VSL = linear speed; VAP = mean trajectory speed. GC1-TRIS 2.5%GOGD = TRIS + 2.5% egg yolk *Gallus gallus domesticus* (Group control); TRIS 2.5% GONM = TRIS + 2.5% egg yolk *Numida meleagris*; TRIS 5% GONM = TRIS + 5% egg yolk *N. meleagris*; TRIS 10% GONM = TRIS + 10% egg yolk *N. meleagris*; TRIS 15% GONM = TRIS + 15% egg yolk *N. meleagris*; TRIS 20% GONM = TRIS + 20% egg yolk *N. meleagris*.

No significant differences were observed (P > 0.05) among the experimental groups and the control groups for the parameters TM, VSL and VAP. For the VCL parameter, the 15% and 20% GONM concentrations gave the best results.

Table 5 shows the effects of sperm cryopreservation in goat semen cryopreserved in ACP-101c® medium and different GONM and GOGD concentrations, by the computerized analysis system, for the parameters LIN, STR and OSC.

**Table 5:** Means (± s.d.) of sperm kinetic parameters (LIN, STR and OSC) in goat semen cryopreserved in ACP-101c® added with different concentrations of GONM evaluated by CASA after thaw.

**Experimental Group**	**LIN (%)**		**STR (%)**	**OSC (%)**
GC2-ACP® 2.5%GOGD		73.92 ± 9.38^ab^	86.31 ± 5.02ª	85.49 ± 6.97ª
ACP® 2.5% GONM	68.91 ± 10.76ª		84.47 ± 7.45ª	81.81 ± 7.05ª
ACP® 5% GONM	72.89 ± 9.54^ab^		85.59 ± 5.02ª	85.15 ± 7.33ª
ACP® 10% GONM	73.89 ± 7.49^ab^		84.54 ± 6.23ª	85.00 ± 5.37ª
ACP® 15% GONM	77.13 ± 7.83^b^		87.64 ± 3.48ª	87.62 ± 6.21ª
ACP® 20% GONM	74.71 ± 11.95^ab^		86.61 ± 6.58ª	85.69 ± 8.97ª

Different letters among columns indicate significant statistical differences (P<0.05). LIN = linearidade; STR = straightness; OSC = oscilation index. GC2-ACP® 2.5%GOGD = ACP-101c® + 2.5% egg yolk *Gallus gallus domesticus* (Group control); ACP® 2.5% GONM = ACP-101c® + 2.5% egg yolk *Numida meleagris*; ACP® 5% GONM = ACP-101c® + 5% egg yolk *N. meleagris*; ACP® 10% GONM = ACP-101c® + 10% egg yolk *N. meleagris*; ACP® 15% GONM = ACP-101c® + 15% egg yolk *N. meleagris*; ACP® 20% GONM = ACP-101c® + 20% egg yolk *N. meleagris*.

The semen cryopreservation in medium containing ACP-101c® at 2.5%, 5%, 10%, 15% and 20% de GONM concentrations gave good results for LIN, STR and OSC and did not differ from the control group (P > 0.05). However, there was significant difference between the 2.5% and 15% groups (P < 0.05).

Table 6 shows the effect of sperm cryopreservation on goat semen cryopreservation in TRIS and different GONM and GOGD concentrations (control group), by computerized analysis system, for the parameters LIN, STR and OSC.

**Table 6:** Means (± s.d.) of sperm kinetic parameters (LIN, STR and OSC) in goat semen cryopreserved in TRIS added with different concentrations of GONM evaluated by CASA post-thaw.

**Experimental Group**	**LIN (%)**	**STR (%)**	**OSC (%)**
GC1-TRIS 2.5%GOGD	7392 ± 9.38^ab^	86.31 ± 5.02ª	85.49 ± 6.97ª
TRIS 2.5% GONM	68.91 ± 10.76ª	84.47 ± 7.45ª	81.81 ± 7.05ª
TRIS 5% GONM	72.89 ± 9.54^ab^	85.59 ± 5.02ª	85.15 ± 7.33ª
TRIS 10% GONM	73.89 ± 7.49^ab^	84.54 ± 6.23ª	85.00 ± 5.37ª
TRIS 15% GONM	77.13 ± 7.83^b^	87.64 ± 348ª	87.62 ± 6.21ª
TRIS 20% GONM	74.71 ± 11.95^ab^	86.61 ± 6.58ª	85.69 ± 8.97ª

Different letters among columns indicate significant statistical differences (P<0.05). LIN = linearidade; STR = straightness; OSC = oscilation index. GC1-TRIS 2.5% GOGD = TRIS + 2.5% egg yolk *Gallus gallus domesticus* (grupo controle); TRIS 2.5% GONM = TRIS + 2.5% egg yolk *Numida meleagris*; TRIS 5% GONM = TRIS + 5% egg yolk *N. meleagris*; TRIS 10% GONM = TRIS + 10% egg yolk *N. meleagris*; TRIS 15% GONM = TRIS + 15% egg yolk *N. meleagris*; TRIS 20% GONM = TRIS + 20% egg yolk *N. meleagris*.

After thawing, the TM assessed by CASA was similar (P > 0.05) in terms of groups tested, therefore there was no effect of the GONM concentration on either dilutant tested. The same was observed for the parameters VSL, VAP, STR and OSC. VCL was superior with the use of 20% GONM in the ACP-101c® dilutant but in TRIS only in comparison to the use of 15% of this protector (P < 0.05), but these groups did not differ from the others (P > 0.05).

In the groups using ACP-101c® as dilutant, it was observed that LIN was lower with the use of 2.5% GONM only when compared with the use of 15% GONM (P < 0.05). The groups using TRIS as dilutant, the use of 20% GONM gave a higher percentage of LIN only when compared to the control group used GOGD (P<0.05).

3.4. Post-thaw sperm viability analysis using the immunofluorescence technique

Table 7 shows the data referent to the sperm viability analyses, using fluorescent probes in cryopreserved goat semen in ACP-101c® and TRIS dilutants.

**Table 7:** Means (± d.p.) of plasmatic membrane integrity mitochondrial activity and acrosome integrity in goat semen cryoprotectant in ACP-101c® and TRIS with the addition of different GONM concentrations assessed by fluorescence staining post-thawing.

**Experimental Group**	**Integral membrane (%)**	**Mitochondrial Activity (%)**	**Integral Acrossome (%)**
G.C.2 ACP® GOGD 2.5%	42.80 ± 17.94	34.40 ± 22.74	43.13 ± 16.73
G.E. ACP® GONM 2.5%	39.40 ± 24.09	37.67 ± 24.42	47.33 ± 21.00
G.E. ACP® GONM 5%	40.93 ± 17.54	43.27 ± 18.05	49.40 ± 20.72
G.E. ACP® GONM 10%	41.80 ± 21.23	36.47 ± 21.42	48.73 ± 24.05
G.E. ACP® GONM 15%	47.29 ± 24.48	47.36 ± 27.42	50.14 ± 16.26
G.E. ACP® GONM 20%	44.60 ± 22.03	35.20 ± 20.18	47.00 ± 26.10
G.C.1 TRIS GOGD 2.5%	39.57 ± 15.61	32.21 ± 14.68	45.86 ± 19.87
G.E. TRIS GONM 2.5%	43.40 ± 21.98	29.60 ±21.06	54.07 ± 19.27
G.E. TRIS GONM 5%	40.33 ± 16.11	38.73 ± 16.87	53.73 ± 23.06
G.E. TRIS GONM 10%	40.53 ± 23.01	50.07 ± 25.80	51.00 ± 12.99
G.E. TRIS GONM 15%	38.53 ± 20.88	40.27 ± 22.00	47.13 ± 11.58
G.E. TRIS GONM 20%	45.87 ± 22.27	44.60 ± 23.73	51.87 ± 27.65

Different letters among columns indicate significant statistical differences (P<0.05). Significant statistical differences were not observed (P>0.05) between lines in the same column. G.C2. ACP®2.5% = Control group containing 2.5% of egg yolk of *Gallus gallus domesticus.* G.E. ACP®2.5%= Experimental group containing 5% of egg yolk of *Numida meleagris* (GOGD). G.E. ACP®5%= Experimental group containing 5% of egg yolk of *Numida meleagris* (GONM). G.E. ACP®10%= Experimental group containing 10% of egg yolk of *Numida meleagris* (GONM). G.E. ACP®15%= Experimental group containing 15% of egg yolk of *Numida meleagris* (GONM). G.E. ACP®20%= Experimental group containing 20% of egg yolk of *Numida meleagris* (GONM). G.C.1 TRIS 2.5% = Control group containing 2.5% of egg yolk of *Gallus gallus domesticus* (GOGD). G.E. TRIS 2.5%= Experimental group containing 5% of egg yolk of *Numida meleagris* (GONM). G.E. TRIS 5%= Experimental group containing 5% of egg yolk of *Numida meleagris* (GONM). G.E. TRIS 10%= Experimental group containing 10% of egg yolk of *Numida meleagris* (GONM). G.E. TRIS 15%= Experimental group containing 15% of egg yolk of *Numida meleagris* (GONM). G.E. TRIS 20%= Experimental group containing 20% of egg yolk of *Numida meleagris* (GONM).

In the fluorescent probe analysis, no differences were observed between the control group and the experimental groups regarding the egg yolk origin or percentage when the ACP-101c® dilutant was used in goat semen cryopreservation (P > 0.05), resulting in integral plasmatic membranes, high mitochondrial activity and integral acrosomes.

In the fluorescent staining test (integral membrane, mitochondrial activity and integral acrosome) no difference was found between the control group and the experimental groups when the TRIS dilutant was used with different GONM percentages (2.5%; 5.0%; 10.0%; 15.0%; and 20.0%) (P > 0.05).

After sperm thawing, it was observed that the plasmatic membrane and acrosome integrity assessed by fluorescence microscopy were equally preserved in all the groups tested in the present study. The same was observed for sperm mitochondrial activity in all the groups post-thawing.

It can be inferred from the sperm viability results assessed by fluorescence that GONM has qualities that make it satisfactory for the process of cryoprervation of goat semen.

### Morphological evaluation of post-thaw sperm cells

Table 8 presents the results of the analysis of the sperm morphology of the semen cryopreserved in ACP-101c® and TRIS with the addition of different GONM concentrations, showing the percentages of sperm with normal morphology, those with bigger defects, smaller defects and with total defects.

**Table 8:** Means (± standard deviation) of the morphology of goat spermatozoa cryopreserved in ACP-101c® added with different concentrations of GONM evaluated after thawing.

**Experimental Group**	**Major Defects (%)**	**Minor Defects (%)**	**Total Defects (%)**	**Sperm** **norma (%)**
GC2-ACP® 2.5%GOGD	2.20 ± 1.99	3.80 ± 1.58	6.00 ± 2.56	88.00 ± 5.11
ACP® 2.5% GONM	2.07 ± 2.06	2.83 ± 1.60	4.90 ± 3.31	90.20 ± 6.61
ACP® 5% GONM	2.77 ± 2.58	5.30 ± 3.62	8.07 ± 4.90	83.87 ± 9.79
ACP® 10% GONM	2.03 ± 1.37	4.07 ± 1.71	6.10 ± 2.44	87.80 ± 4.87
ACP® 15% GONM	1.77 ± 1.53	3.47 ± 1.63	5.23 ± 1.94	89.53 ± 3.87
ACP® 20% GONM	1.57 ± 1.00	3.50 ± 1.76	5.07 ± 1.36	89.87 ± 2.72
GC1 TRIS GOGD 2.5%	2.30 ± 1.75	2.47 ± 1.48	4.77 ± 2.10	90.47 ± 4.19
G.E. TRIS GONM 2.5%	2.13 ± 1.27	2.90 ± 1.56	5.03 ± 2.42	89.93 ± 4.83
G.E. TRIS GONM 5%	2.53 ± 2.37	3.10 ± 1.64	5.63 ± 2.90	88.73 ± 5.80
G.E. TRIS GONM 10%	2.43 ± 1.80	3.87 ± 2.47	6.30 ± 3.40	87.40 ± 6.80
G.E. TRIS GONM 15%	2.20 ± 2.16	4.33 ± 3.07	6.53 ± 3.73	86.93 ± 7.45
G.E. TRIS GONM 20%	1.60 ± 1.53	3.13 ± 2.47	4.73 ± 3.29	90.53 ± 6.59

Different letters among columns indicate significant statistical differences (P<0.05). Significant statistical differences were not observed (P>0.05) between lines in the same columnG.C.2 ACP®2.5% = Control group containing 2.5% of egg yolk of *Gallus gallus domesticus.* G.E. ACP®2.5%= Experimental group containing 5% of egg yolk of *Numida meleagris* (GOGD). G.E. ACP®5%= Experimental group containing 5% of egg yolk of *Numida meleagris* (GONM). G.E. ACP®10%= Experimental group containing 10% of egg yolk of *Numida meleagris* (GONM). G.E. ACP®15%= Experimental group containing 15% of egg yolk of *Numida meleagris* (GONM). G.E. ACP®20%= Experimental group containing 20% of egg yolk of *Numida meleagris* (GONM). G.C.1 TRIS 2.5% = Experimental group containing 2.5% of egg yolk of *Gallus gallus domesticus* (GOGD). G.E. TRIS 2.5%= Experimental group containing 5% of egg yolk of *Numida meleagris* (GONM). G.E. TRIS 5%= Experimental group containing 5% of egg yolk of *Numida meleagris* (GONM). G.E. TRIS 10%= Experimental group containing 10% of egg yolk of *Numida meleagris* (GONM). G.E. TRIS 15%= Experimental group containing 15% of egg yolk of *Numida meleagris* (GONM). G.E. TRIS 20%= Experimental group containing 20% of egg yolk of *Numida meleagris* (GONM).

According to the results obtained, both the control group and the experimental groups preserved the sperm morphology in the freeze-thaw process when the TRIS dilutant was used (P > 0.05).

## Discussion

The pH found (6.99) favors sperm survival (Purdy, 2006). The moisture and ash contents were similar; GONM and GOGD (Bashir et al., 2015).

The lipid value of GONM (34.56%) was higher than that of GOGD and could lead to a bigger protective action of the sperm membrane during the freeze/thaw process (Watson, 1995).

Regarding the egg yolk bromatological analysis, there is still little information on the nutritional qualities of eggs from domestic and wild birds (Bashir et al., 2015). Studies on the composition of *Gallus gallus domesticos* eggs are well reported in the literature. However, such information has not been as well reported for other bird species, including the *Numida meleagris*.

According to the literature (Bashir et al., 2015), egg yolk composition assessed in three different species, *Gallus gallus domesticus*, *Numida meleagris* and *Gallus gallus hibrido* showed no significant difference in the protein content (3.34; 4.40 and 3.85%) and fat content (27.65; 31.49 and 30.41), respectively. However, the results for these two parameters, in the present study, were higher (9.37% and 34.56%, respectively) than those reported by the authors quoted above. Nevertheless, they were similar to values found by (Adeyeye, 2010), who reported 15.74% protein and 31.91% fat contents.

It is pointed out that in spite of the risk of transmission of diseases of animal origin egg yolk added to seminal dilutants (Silva et al., 2006), is still the extracellular cryoprotectant of choice for semen cryoprotectant (Anand et al., 2017; Narwade et al., 2017).

Several studies have shown satisfactory results regarding substituting *Galus galus domesticus* egg yolk with egg yolk of other species, as, for example *Alectoris chukar* (pheasant) egg yolk, that favored an improvement in total motility and progressive motility (Hunes and Webb, 2006) and *Anas platyrhynchos domesticus* (Duck) egg yolk, that favored an increase in motility and a reduction in defective spermatozoids (Andrabi et al., 2008), and *Rhea americana* (Ema) aq, that was shown to be a viable alternative in increasing semen quality post-thawing. But, to date, there are no studies assessing the efficaciousness of *Numida meleagris* egg yolk in goat semen cryopreservation.

The motility values in the present study were similar to those observed by (Dorado et al., 2007) when skim milk and TRIS were used as base dilutants, with the addition of 20% *Galus galus domesticus* egg yolk, in goat semen cryopreservation.

The sperm motility results at 5 minutes post-thawing, quantified in the present study, were similar to those obtained by other authors who cryopreserved goat semen in TRIS-based dilutants with *Galus galus domesticus* egg yolk (Amidi et al., 2010; Hidalgo et al., 2006).

Several studies have reported low values in the goat species for post-thawing progressive motility: 22 % in native and 6% in British Alpine goats (Câmara et al., 2019). This is a very important sperm parameter in reproducer choice and also in the assessments of semen freezing protocols (Câmara et al., 2019; Santiago-Moreno et al., 2017).

Previous studies (Bispo et al., 2011) assessed by *in vitro* tests and *in vivo* fertility which concentrations of *Galus galus domesticus* egg yolk (low, 2.5% or high 20%) in the glucose dilutant EDTA presented better frozen goat semen preservation. After thawing, the dilutant with 2.5% *Galus galus domesticus* egg yolk improved the post-thawing sperm parameters, regardless of the reproductive season.

The results of the present study suggest that substituting the cryoprotectant *Galus galus domesticus* egg yolk (GOGD) with *Numida meleagris* egg yolk(GONM) was shown to be a viable alternative, as it maintained the sperm parameters at levels similar to those reported with use of *Galus galus domesticus* egg yolk.

*Gallus gallus domesticus* egg yolk is a constituent routinely used in seminal cryopreservation of several species and presents many advantages, by conferring protection to spermatozoids against heat shock caused during the cryopreservation process (Layek et al., 2016; Oliveira et al., 2011). In this sense, the results obtained in the present study indicated that *Gallus gallus domesticus* egg yolk can be satisfactorily substituted with *Numida meleagris* egg yolk for this purpose.

The TM percentages found both in the samples cryopreserved in TRIS and ACP-101c®, at the different GONM concentrations, were lower than those reported by Oliveira et al. (2011), as the TRIS dilutant was used and better results were obtained (TM = 54.5±16.5%; MP = 27.1±11.3%; VAP = 55.6±10.6 µm/s; and VSL = 47.9±10.5 µm/s) when compared to the ACP-101c dilutant . On the other hand, after incubation at 37 ºC for five minutes, the kinetic parameters of the spermatozoids cryopreserved in ACP-101c® medium showed the potential of this dilutant in seminal cryopreservation of goat reproducers.

VCL and VSL did not surpass 100 μm/s in the experimental groups. In this case, it is known that if this parameter surpasses 250 μm/s, it indicates that there was spermatozoid hyperactivation and this hyperactivation is a restricted characteristic, since it is sperm activation in the proximities of where fertilization occurs, that is, in the female reproductive tract, specifically in the ampoule of the uterine tube. As described in the literature (Verstegen et al., 2002), this premature activation will reduce the useful spermatozoid life, leading to a lower chance of fertilization.

In other studies using ACP® as cryopreservation medium with the addition of egg yolk did not show statistical difference regarding the post-thawing seminal quality (Câmara et al., 2019).

In other studies using ACP®, it was observed that the plasmatic membrane and acrosome integrity, assessed by fluorescence microscopy as cryopreservation medium with the addition of hen egg yolk, also did show not statistical differences regarding post-tawing seminal quality (Silva et al., 2006).

Egg yolk is an important component in refrigeration and freezing media to protect semen of several species, and its properties were discovered in 1939 (Bathgate et al., 2006).

The main post-thawing morphological alterations found were related to head and tail aterations. In addition, most of the head alterations observed post-thawing of the semen were concentrated in the acrosome. Similar results have been reported in several studies, that describe the deleterious effects of cryopreservation on the percentage of acrosome abnormalities in goat spermatozoids (Azerêdo et al., 2001; Ferrari and Barnabe, 1999; Hidalgo et al., 2006; Oliveira et al., 2011).

## Conclusion

*Numida meleagris* egg yolk, as an external membrane cryoprotectant added to ACP-101c® and TRIS extenders, did not impair the sperm viability of goat semen. In addition, through this research, it was possible to attest that the new diluents (TRIS + Numida meleagris egg yolk and ACP-101c + Numida meleagris egg yolk) were as satisfactory as the already established and widely used diluents in scientific studies (TRIS + Galus gallus domesticus and ACP-101c egg yolk + Galus gallus domesticus) regarding the maintenance of sperm cell quality. Thus, the diluents tested in this study can be used in practice, being an innovation for biotechnologies applied to goat semen, favoring goat farming.
